# S-PRG-based composites erosive wear resistance and the effect on surrounding enamel

**DOI:** 10.1038/s41598-021-03745-3

**Published:** 2022-01-17

**Authors:** Bianca Tozi Portaluppe Bergantin, Camilla Cristina Lira Di Leone, Thiago Cruvinel, Linda Wang, Marília Afonso Rabelo Buzalaf, Alessandra Buhler Borges, Heitor Marques Honório, Daniela Rios

**Affiliations:** 1 Department of Pediatric Dentistry, Orthodontics and Public Health, Bauru School of Dentistry, University of São Paulo, Bauru, SP Brazil; 2 Department of Operative Dentistry, Endodontics and Dental Materials, Bauru School of Dentistry, University of São Paulo, Bauru, SP Brazil; 3 Biochemistry Department, Bauru School of Dentistry, University of São Paulo, Bauru, SP Brazil; 4 Department of Restorative Dentistry, Institute of Science and Technology, São Paulo State University-UNESP, São Paulo, Brazil

**Keywords:** Biochemistry, Health care, Materials science

## Abstract

This study evaluated Surface Pre-Reacted Glass-ionomer (S-PRG)-based-composites’ surface resistance against erosive wear and their protective effect on surrounding enamel. Bovine enamel was randomized into 12 groups (n = 10/group) [erosion (e) or erosion + abrasion (a)]: nanohybrid-S-PRG-based composite (SPRGe/SPRGa), nanohybrid-S-PRG-based bulk-fill (SPRGBFe/SPRGBFa), nanoparticle-composite (RCe/RCa), nanohybrid-bulk-fill (BFe/BFa), Glass Hybrid Restorative System (GHRSe/GHRSa), and resin-modified glass-ionomer-cement (RMGICe/RMGICa). Cavities were prepared and restored. Initial profile assessment was performed on material and on adjacent enamel at distances of 100, 200, 300, 600, and 700 μm from margin. Specimens were immersed in citric acid (2 min; 6×/day for 5 days) for erosion. Erosion + abrasion groups were brushed for 1 min after erosion. Final profile assessment was performed. Two-way ANOVA and Tukey-test showed: for erosion, the GHRSe and RMGICe presented greater material wear compared to the other groups (p = 0.001); up to 300 μm away from restoration, GHRSe and SPRGBFe were able to prevent enamel loss compared to RMGICe and other composite groups (p = 0.001). For erosion + abrasion, none of the materials exhibited a significant protective effect and S-PRG-based groups showed lower wear than RMGICa and GHRSa, and higher wear than composites (p = 0.001). S-PRG-based-composites can diminish surrounding enamel loss only against erosion alone, similarly to GIC, with advantage of being a more resistant material.

## Introduction

Erosive tooth wear is the loss of tooth tissue due to a chemical–mechanical process, in which extrinsic (dietary) and intrinsic (gastric) acids interact with attrition and/or abrasion^1^. The prevalence of this alteration is high and seems to be increasing^2–4^. The ideal treatment for erosive tooth wear is based on early diagnoses and implementation of non-operative management strategies, acting on the risk factors for its development over time^5,6^. When risk factors are not effectively controlled, enamel, and eventually dentine, is lost. According to the Radboud philosophy, even for patients with severe tooth wear, counselling and monitoring is the treatment of choice when there is no complaint^7^. Nevertheless, restorative treatment is recommended when there is loss of the vertical dimension of occlusion, pain, and/or esthetic issues. In these cases, minimally invasive and adhesive restorative strategies are indicated^7^.

The longevity of restorative materials under erosive and abrasive challenges depends on durability of the material and of the interface between dental tissue and restoration^8–10^. In general, glass-ionomer cements (GICs) are more susceptible to wear than composites under chemical–mechanical challenges^11^, but only GICs release fluoride, which could enhance the acid resistance of the dental tissue adjacent to restorations^12,13^. Therefore, there is no ideal material with both benefits of resistance and preventive effects against erosive tooth wear.

The Giomer's technology was developed to enhance material properties, providing wear resistance associated to fluoride release^14^. This technology is based on Surface Pre-Reacted Glass-ionomer (S-PRG) fillers that are synthesized by the reaction between fluoro-boro-aluminosilicate glass and a polyacrylic acid solution^14^. This filler was shown to release multiple ions including F^−^, Sr^2+^, Na^+^, BO_3_^3−^, Al^3+^, and SiO_3_^2–16^, which results in the buffering of lactic acid solution^17^ and prevents enamel demineralization around the material^18–23^. The S-PRG filler is used in various dental materials including composite resins^24^. The buffering effect and the potential to prevent demineralization might be interesting characteristics of a material to control enamel loss around restorations in patients with risk factors for erosive tooth wear. There is only one study in the Literature regarding this topic, showing that S-PRG-based composites presented minimal surface wear, like regular composite resins when subjected to erosion and an intermediate wear, between resins and glass ionomer cements when subjected to erosion and abrasion^25^. In addition, S-PRG-based composites were not able to prevent enamel wear near the restoration^25^. However, it is important to confirm these results with different types of S-PRG-based composites and at different distances in relation to the restoration margin, to verify the limit of the possible preventive effect of the material. Therefore, this in vitro study aimed to evaluate the performance of S-PRG-based composite resins when submitted to erosion and abrasion processes and their influence on the adjacent enamel at different distances. The effects were compared to those of GICs (with fluoride-release properties) and resin composite (with acid-resistance properties). The null hypotheses were that: (1) there is no difference in the resistance to erosive and/or abrasive challenges between S-PRG-based composites and resin composites; and (2) there is no difference in the effect between S-PRG-based composites and GICs on adjacent dental tissue, when enamel is subjected to erosion with or without abrasion.

## Methods

### Experimental design

All experimental protocols were approved by the Research Ethics Committee of the Bauru School of Dentistry-University of São Paulo (FOB/USP) (protocol number 88429518.2.0000.5417).

The factors under study were type of material (six levels) and wear condition (two levels). A sample size of 10 blocks per group was estimated considering a α error of 5%, β error of 20%, 2.6 µm standard deviation (pilot study) and a minimum detectable difference in the means of 5 µm enamel loss. One hundred and twenty crowns of bovine teeth composed the sample. The specimens were randomly assigned into 12 groups (n = 10 per group): SPRGe and SPRGa (nanohybrid-S-PRG-based composite resin—Beautifil II); SPRGBFe and SPRGBFa (nanohybrid-S-PRG-based bulk-fill composite resin—Beautifil Bulk Restorative); CRe and CRa (nanoparticle-composite resin—Filtek Z350 XT); BFe and BFa (nanohybrid-bulk-fill composite resin—Filtek Bulk Fill); GHRSe and GHRSa (glass hybrid restorative system—EQUIA Forte); and RMGICe and RMGICe (resin-modified glass-ionomer cement—Riva). The groups with the letter “e” were subjected to erosion and those with the letter “a”, to erosion + abrasion.

Circular cavities were prepared on each specimen and then restored with the different materials according to manufacturers’ instructions (Table 1). During 5 days, erosion was simulated in vitro (‘e’ Groups) by placing the specimens in a 0.5% citric acid solution for 2 min, 6 times per day. For groups subjected to erosion associated with abrasion (‘a’ Groups), each erosive challenge was followed by abrasion using a toothbrush machine with fluoridated dentifrice:water slurry (3:1) for 60 s. Between challenges, the blocks were kept in artificial saliva^26^. The response variable was material and enamel loss measured by profilometry.

**Table 1:** Composition of each material and manufacturers’ instructions.

Material/lot/color	Group	Composition	Application steps
Beautifil II Lot 051829/Color A2 (Beautiful II; Shofu, Kyoto, Japan)	S-PRG-based composite resin (SPRG)	Bis-GMA, TEGDMA, multifunctional filler, S-PRG filler based on F-Br-Al-Si glass	37% phosphoric acid^a^ (15 s), rinsing with water for 15 s and blot dry on tissue paper, Universal Adhesive^b^ (20 s), drying with air jet (5 s), light curing^c^, insertion of the material and light curing (40 s)
Beautifil Bulk Restorative Lot 031828/Color Universal (Beautiful Bulk Restorative; Shofu, Kyoto, Japan)	S-PRG-based bulk-fill resin (SPRGBF)	Bis-GMA, UDMA, Bis-MPEPP, TEGDMA, S-PRG based on F-Br-Al-Si glass	
Filtek One Bulk Fill Lot N963374/Color A2 (FIltek One Bulk Fill; 3M, Sumaré, Brazil)	Bulk-fill composite resin (BF)	AFM, DDDMA, UDMA, AUDMA, pocrylat resins, ytterbium trifluoride, zirconia/silica cluster	
Filtek Z350 XT Lot 1710900734/Color A2 Dentin (FIltek Z350; 3M-ESPE, Sumaré, Brazil)	Composite resin (CR)	Bis-GMA, Bis-EMA, UDMA, TEGDMA, zirconia/silica cluster and silica nanoparticle	
EQUIA Forte Lot 1709191 (Equia Forte; GC America, Costa Mesa, USA)	Glass hybrid restorative system (GHRS)	Powder: F–Al–Si glass, Polyacrylic acid powder, pigment. Liquid: polyacrylic acid, distilled water, polybasic carboxylic acid	26% polyacrylic acid^e^ (10 s), rinsing with water for 10 s and blot dry on tissue paper, capsule agitation (10 s), material application, chemical polymerization (3 min), protector application^d^ and light curing (20 s)
RIVA Light Cure Lot J1602181EG (RIVA; SDI, Victoria, Australia)	Resin-modified glass ionomer cement (RMGI)	Powder: F–Al–Si glass. Liquid: polyacrylic acid, HEMA and tartaric acid	26% polyacrylic acid^e^ (10 s), rinsing with water for 10 s and blot dry on tissue paper, capsule agitation (10 s), material application, light curing^c^ (20 s)

*Bis-GMA* bisphenol A-glycidyl methacrylate, *TEGDMA* triethylene glycol dimethacrylate, *F* fluoride, *Br* boron, *Al* aluminium, *Si* silicate, *UDMA* Urethane Dimethacrylate, *Bis-MPEPP* Bisphenol-A polyethoxy-dimethacrylate, *AFM* addition-fragmentaion monomers, *DDDMA* 1,12-dodecanediol dimethacrylate, *AUDMA* Aromatic urethane dimethacrylate, *HEMA* 2-hydroxyethyl methacrylate.

^a^Condac 3; FGM, Joinville, Brazil/Lot 1301317.

^b^Adper Single Bond Universal; 3 M-ESPE, Sumaré, Brazil/Lot 643238.

^c^Optilight MAX; Dabi Atlante, Ribeirão Preto, Brazil/LED 1200 mW/cm ^2^.

^d^Equia Forte Coat Protector; GC America, Costa Mesa, USA/Lot 1702081.

^e^RIVA Conditioner; SDI, Victoria, Australia/Lot 170705.

### Specimen preparation

Approximately 140 bovine teeth were used in the present study. First, the roots were separated from their crowns using a cutting machine (National Factory; Nevoni Single-phase Motors, São Paulo, Brazil) and a Diaflex-F diamond disc (Wilcos do Brasil; Indústria e Comércio, Petrópolis, Brazil). Crowns were individually placed in a cylindrical silicone mold (inner radius of 2.8 cm) and embedded in acrylic resin (Jet; Artigos Odontológicos Classico Campo Limpo Paulista, Brazil). Then, the specimens were ground flat with water-cooled silicon carbide discs of 600 and 1200 grit (Buehler; Illinois Tool Works, IL, USA) and polished with felt disc wetted with 1-µm diamond spray (Buehler; Illinois Tool Works, IL, USA). The enamel specimens were ultrasonicated (Ultrasonic Cleaner Mod USC 750; Unique Ind. And Com, São Paulo, Brazil) in deionized water for 2 min between the polishing steps.

A profile assessment was performed in each specimen to ascertain the planning and 120 specimens were selected. The profile was obtained with contact profilometer (MarSurf GD 25; Mahr, Göttingen, Germany), coupled to a computer with a MarSurf XCR 20 contour software. Then, the specimens were randomly distributed into 12 groups of 10 using a spreadsheet program (Microsoft Excel; Microsoft, Redmond, USA) with the "RANDOM" function of the mathematical category.

Circular cavities were prepared at the center of the crown using #2096 cylindrical diamond bur (KG Sorensen; KG Sorensen, São Paulo, Brazil), with a diameter of 1.4 mm. A custom-made automatic device was used to standardize the depth of preparation (1.5 mm). For the composite with and without S-PRG fillers, 37% phosphoric acid and adhesive system were applied. Each material was handled according to the manufacturers’ instructions (Table 1). All restorative materials were inserted in a single increment in the prepared cavity and the surface of the restorative material was covered with a polyester strip and a glass slab under pressure to expel excess material from the cavity, avoiding too many surface irregularities and presence of bubbles.

After 7 days of storage at 37 °C in 100% relative humidity, the restorations were polished with water-cooled silicon carbide discs as described before.

### Initial profilometric analysis

Enamel blocks were marked with a scalpel blade (Prime Cirurgica; Embramac, Itapira, Brazil) at two opposite sites with a distance of 0.3 mm from the margin of the restoration, resulting in two reference areas with 1.0 mm (at the border) and a test area with 2.0 mm, containing the restoration (at the center). Subsequently, initial surface profiles were obtained using a profilometer (MarSurf GD 25; Mahr, Göttingen, Germany) and a contour software (MarSurf XCR 20; Mahr, Göttingen, Germany), aiming to read the surface contour for posterior overlap and measurement of the wear. To standardize their position, specimens were fixed to a special holder and their locations were recorded allowing their exact replacement after the erosive–abrasive challenges. To analyze restorative material loss, two surface profiles were obtained through scanning from the reference to the test area, at the center of the restoration with a distance of 100 μm between them. To analyze loss of enamel adjacent to the restoration, five profiles were obtained by scanning from the reference to the test area, at 100, 200, 300, 600, and 700 μm distances from the restoration margin.

Then, the previously demarcated reference areas were protected with nail polish (Colorama Maybelline; Cobra Cosméticos, São Paulo, Brazil) during erosive and/or abrasive challenges.

### In vitro erosive and abrasive challenges

All the specimens were subjected to six daily erosive cycles for 5 days by being immersed, for 2 min, in 30 mL of citric acid prepared in laboratory, at a dilution of 0.5%, with pH 2.5, simulating an acidic diet under agitation in circle movements at 50 cycles per minute and at a controlled temperature of 25 °C. After erosion, half of the specimens (erosion groups) were rinsed with deionized water for 5 s and kept in artificial saliva (0.33 g KH_2_PO_4_, 0.34 g Na_2_HPO_4_, 1.27 g KCl, 0.16 g NaSCN, 0.58 g NaCl, 0.17 g CaCl_2_, 0.16 g NH_4_ Cl, 0.2 g urea, 0.03 g glucose, 0.002 g ascorbic acid, pH 7)^26^ for 2 h, until the next daily cycle, and overnight (14 h), at 37 °C.

The other half of the specimens (erosion + abrasion groups) was submitted to toothbrush abrasion after each erosive challenge, (6 times per day for 5 days). Extra-soft toothbrushes (Colgate Twister; Colgate Palmolive Industrial, São Bernardo do Campo, Brazil) were personalized for each specimen and fixed parallel to the dental surface on a brushing machine (Máquina de escovação; Biopdi, São Carlos, Brazil). The dentifrice slurry was prepared daily by diluting fluoride dentifrice (Colgate triple action; Colgate-Palmolive Industrial, São Bernardo do Campo, Brazil) in distilled water in a 1:3 ratio (weight-volume, according to ISO 14569-1), and agitated before each use. The slurry was automatically dropped on each specimen (≈ 3 mL). Each abrasive cycle consisted of brushing the specimens for 60 s with 100 reciprocal linear motion (back and forth) and force of 250 g, at temperature of 37.5 °C. After abrasion, specimens were rinsed with deionized water for 5 s and kept immersed in artificial saliva similarly to the erosion groups.

### Profilometric analysis

After the in vitro erosive and abrasive cycles, the nail polish was removed from the reference areas and the profilometric analysis was performed at the same sites of the initial measurements. Baseline and final profiles were perfectly matched, since the enamel specimens could be precisely repositioned in the profilometer wells. The material and enamel losses were quantitatively determined using a specific software (MarSurf XCR 20; Mahr, Göttingen, Germany) by calculating the vertical difference (average depth of the surface) between baseline and final surface profiles. The material loss corresponded to the average value of the two profiles made at the center of the material (in micrometers). The enamel loss was analyzed in each evaluated distance from the restoration margin.

### Statistical analysis

The assumptions of equality of variances and normal distribution of errors were met. Two-way ANOVA and Tukey test were applied to compare the differences of materials and enamel losses among groups. The significance level was 5% and the software used was Sigma Plot for Windows (Sigma Plot; Systat Software, San Jose, USA).

## Results

Table 2 shows the results for material loss. A significant difference was found for type of material (p < 0.001), condition (p < 0.001), and their interaction was also significant (p < 0.001). Among the groups subjected to the erosion condition, the composite groups (CRe, BFe) and the S-PRG-based groups (SPRGe, SPRGBFe) presented statistically similar material loss (p > 0.999), which was less than that of the GIC groups (GHRSe, RMGICe) (p < 0.001). Among those of the erosion + abrasion condition, the composite groups (CRa, BFa) presented significantly less material loss, followed by S-PRG-based composite (SPRGa, SPRGBFa) and then by the GIC groups (GHRSa, RMGICa), with statistical difference among them (p < 0.001). Considering each material, both GICs and both S-PRG-based composites showed statistically significant higher material loss with erosion + abrasion in comparison to erosion alone (p < 0.001). The composite groups presented statistically similar material loss when subjected to erosion and erosion + abrasion conditions (p = 1).

**Table 2:** Average wear of material (μm) and standard deviation (± SD) of the studied groups.

Material	Giomer beautifil II	Giomer—beautifil bulk restorative	Z350 XTResin	Bulk fill resin	EQUIA forte	RIVA LC
**Condition**						
ERO	1.7 (± 0.7)^a^	1.3 (± 1.0)^a^	0.6 (± 0.4)^a^	1.1 (± 0.8)^a^	7.5 (± 4.6)^b,c^	11.3 (± 5.5)^c^
ERO + ABR	7.2 (± 1.6)^b,c^	6.7 (± 2.9)^b^	0.9 (± 0.7)^a^	1.5 (± 0.8)^a^	19.9 (± 3.0)^d^	17.9 (± 4.7)^d^

Different letters indicate statistical difference among group and conditions (two-way ANOVA and Tukey test).

Material (p < 0.001), condition (p < 0.001) and significant interaction (p < 0.001).

*ERO* erosion condition, *ERO + ABR* erosion and abrasion condition.

Table 3 shows the results for enamel loss at 100, 200, 300, 600, and 700 µm distances from the restoration margin. There was a significant difference for type of material (p < 0.001), for condition (p < 0.001), and a statistically significant interaction in each distance (p < 0.001). For erosion + abrasion condition, there was no statistically significant difference among materials in relation to enamel wear at all distances (p > 0.001). When considering each material alone, the same behavior between conditions on studied distances were observed. All materials resulted in statistically significant higher enamel loss when erosion was associated with abrasion compared to erosion alone (p < 0.001), except CR (Z350 resin), which did not show any significant difference between both conditions (ERO and ERO + ABR) (p > 0.108).

**Table 3:** Enamel wear (μm) and standard deviation (SD) of the studied groups at 100, 200, 300, 600, and 700 μm from the restoration margin.

Material	Giomer—beautiful II	Giomer—beautiful bulk restorative	Z350 XT resin	Bulk fill resin	EQUIA forte	RIVA LC
**Distance/condition**						
100 µm						
ERO	30.3 (± 3.4)^b,d^	28.3 (± 2.5)^b^	38.3 (± 4.0)^c,e^	35.6 (± 2.8)^d,e^	27.7 (± 1.2)^b^	31.4 (± 2.9)^b,d^
ERO + ABR	44.1 (± 5.2)^a,c^	44.6 (± 5.0)^a^	43.2 (± 4.4)^a,c^	47.1 (± 4.6)^a^	43.7 (± 3.0)^a,c^	47.3 (± 6.7)^a^
200 µm						
ERO	30.6 (± 3.3)^b,c^	29.4 (± 3.6)^b,c^	38.2 (± 4.1)^d,e^	35.0 (± 2.6)^c,e^	27.9 (± 1.4)^b^	32.1 (± 3.0)^b,c,e^
ERO + ABR	44.2 (± 5.1)^a,d^	45.5 (± 6.5)^a^	44.0 (± 3.2)^a,d^	47.1 (± 5.0)^a^	43.9 (± 2.3)^a,d^	47.0 (± 6.9)^a^
300 µm						
ERO	30.9 (± 3.5)^b,c^	29.7 (± 4.5)^b^	37.8 (± 4.0)^c,d,e^	34.4 (± 2.6)^b,c,e^	28.2 (± 1.4)^b^	33.1 (± 2.2)^b,c^
ERO + ABR	44.5 (± 4.7)^a,d^	46.5 (± 11.3)^a^	41.9 (± 4.6)^a,d,e^	46.8 (± 4.7)^a^	44.2 (± 2.0)^a,d^	46.9 (± 8.1)^a^
600 µm						
ERO	30.7 (± 3.2)^b,c^	29.8 (± 4.1)^b,c^	36.3 (± 3.7)^c,d^	33.7 (± 2.6)^b,c^	28.5 (± 1.4)^b^	33.9 (± 2.4)^b,c^
ERO + ABR	43.7 (± 4.8)^a^	43.6 (± 7.3)^a^	41.6 (± 4.5)^a,d^	45.9 (± 4.3)^a^	44.4 (± 2.3)^a^	45.7 (± 7.5)^a^
700 µm						
ERO	30.4 (± 2.8)^b,c^	30.0 (± 3.6)^b,c^	36.1 (± 3.5)^c,d^	33.3 (± 2.2)^b,c^	29.0 (± 1.6)^b^	34.2 (± 2.4)^b,c^
ERO + ABR	43.7 (± 4.2)^a^	43.2 (± 8.2)^a^	41.3 (± 3.9)^a,d^	45.3 (± 5.0)^a^	44.6 (± 2.4)^a^	45.3 (± 6.9)^a^

Different letters indicate statistically significant difference among material and condition at the same distance (two-way ANOVA and Tukey test).

At distance 100 µm: material (p < 0.001), condition (p < 0.001) and significant interaction (p < 0.001).

At distance 200 µm: material (p < 0.001), condition (p < 0.001) and significant interaction (p = 0.001).

At distance 300 µm: material (p = 0.070), condition (p < 0.001) and significant interaction (p = 0.003).

At distance 600 µm: material (p = 0.030), condition (p < 0.001) and significant interaction (p = 0.009).

At distance 700 µm: material (p = 0.080), condition (p < 0.001) and significant interaction (p = 0.008).

*ERO* erosion condition, *ERO + ABRA* erosion and abrasion condition.

When considering only erosion, the distance from the restoration margin affected the enamel loss. At 100 µm, the SPRGBFe (Giomer Beautifil Bulk) and GHRSe (EQUIA) groups had statistically significant less enamel wear than the CRe (Z350) and BFe (Bulk Fill) (p < 0.001), but were statistically similar to SPRGe (Giomer Beautifil II) (p > 0.961) and RMGICe (Riva LC) (p > 0.704); those 2 groups were also statistically similar to CRe (Bulk Fill) (p > 0.002).

At 200 µm from the margin, considering erosion alone, both S-PRG-based composites (S-PRGe-Beautifil and S-PRGBFe-Beautifil Bulk) groups and the conventional GIC (GHRSe-EQUIA) group promoted statistically similar enamel loss (p > 0.957), which was lower when compared to the composite resin (CRe-Z350) group (p < 0.001). The BFe group was statistically similar to the S-PRG-based composite groups (S-PRGe and S-PRGBFe) (p > 0.136). The RMGICe (Riva LC) was statistically similar to both composite groups (CRe and BFe) (p > 0.713).

At 300 µm from the margin, in erosion condition, only the SPRGBFe (Giomer Beautifil Bulk) and the GHRSe (EQUIA) groups resulted in statistically significant less enamel wear than the RCe (Z350) (p < 0.05), but both were statistically similar to SPRGe, RMGICe, and BFe (Giomer Beautifil II, Riva LC and Bulk Fill, respectively) (p > 0.271).

At 600 and 700 µm from the margin, when considering erosion condition, there was no significant difference for enamel loss among the groups, except for the GHRSe (EQUIA) that resulted in less enamel loss compared to CRe (Z350) (p < 0.001).

## Discussion

Many restorative materials have been tested regarding their resistance to erosive tooth wear^11,13,19,22,27,28^. However, in addition to material resistance, an ideal material might also prevent wear around the restoration. For patients who present erosive tooth wear, the ideal management is the association with measures that eliminate the causes of erosive wear^5–8^. However, this approach is not always feasible, and when a restoration is needed, the use of bioactive materials, resistant and capable of protecting the adjacent tooth structure is highly desirable.

Regarding material loss due to acid attack, the composite groups showed the lowest wear, which is in accordance with previous studies^11,29^ and can be explained mostly by the low acid degradation of the composite matrix organic content^11,30^. In the composite groups, material wear was similar after erosion alone or erosion + abrasion. The mechanical resistance of the composite matrix in addition to bond stability between the filler and the matrix increases the abrasion resistance of the composite-based restorative materials^30^.

When considering erosion, the first null hypothesis of the present study was not rejected since the S-PRG-based composite groups showed similar wear compared to the composite groups, which is in line with a previous study^31^. This result can be attributed to the presence of bis-GMA and TEGDMA matrix, which is resistant to acid^24^ and to the high filler content. Contrary to the present study, it was demonstrated that the hardness and roughness of the Giomer (resin composite with S-PRG fillers) are more affected by citric acid than composite resins due to the fluoroborosilicate glass fillers’ greater susceptibility to degradation by weak acids than the zirconia-silicate filler of the conventional composite^24^. For the erosion + abrasion challenge, the S-PRG-based composite resins showed intermediate material wear compared to GIC (higher wear) and composite resins (lower wear). Composites with nanofillers provide a more homogeneous and smoother surface, which is less susceptible to damage by mechanical forces^24^. Although fluorosilicate glass fillers are more superficial and prominent promoting ions release, this characteristic might also facilitate their removal by mechanical forces.

The loss from erosion of the resin-modified GIC was significantly higher than the glass hybrid restorative system, which was designed for load-bearing areas^32^ and contains highly reactive glass particles dispersed within the conventional glass ionomer structure and a high molecular weight polyacrylic acid^33^. Despite the manufacturers’ name suggesting otherwise, this material can be considered a GIC since it is formed by the reaction between a weak polymeric acid and the inorganic glass powder^34^. The loss of material was high for all GICs studied, which is in line with the literature^11,35^. The siliceous hydrogel layer of GICs is easily dissolved by acidic solutions, resulting in peripheral matrix dissolution and exposure of the glass particles^27,36,37^. With matrix dissolution, the material is less resistant to toothbrush abrasion as shown by the present results (Table 1) and in a previous study^27^. When subjected to erosion and abrasion, the studied GICs also showed higher surface loss when compared to resin composites, which was expected as the GIC is well known to lack the excellent values of abrasive wear resistance of the composites^33,38^.

The results showed that the S-PRG-based composite materials similarly to glass ionomer cement were able to decrease enamel loss against erosion only, at 100–300 µm from the restoration margin, when compared to resin composite. For erosion associated to abrasion none of the studied materials were able to protect enamel. Therefore, the second null hypothesis was not rejected. The S-PRG-based bulk-fill composite resin (SPRGBFe-Beautifil bulk fill) reduced by 26% the enamel erosion, which was similar to the glass hybrid restorative system (GHRSe-EQUIA), with a 27% reduction. The reference group for enamel loss reduction was the one filled with composite resin (Z350), as this was the less effective material in protecting against erosive wear, and the amount of enamel loss of this group was considered as 100%. This reduction was significant up to 300 µm for SPRGBFe and GHRSe. Therefore the protective effect on adjacent enamel promoted by S-PRG-based composites, especially at distances up to 300 µm from the restoration, was notable. We hypothesize that fluoro-alumina-borosilicate glass filler (S-PRG) can release ions that neutralize the erosive acids and reduce enamel demineralization^39^. A previous study found that Beautifil II was capable of increasing the pH of solutions up to neutral (6–7) and the authors attributed this ability to the S-PRG fillers^40^. In addition, strontium presents a synergistic effect when associated to fluoride, with the advantage of replacing hydroxyl and calcium ions in the apatite structure^35^, resulting in a more acid-resistant strontium and fluoride-modified apatite that may be less soluble under acid exposure. Only one study found no protective effect of the S-PRG-based composite (Beautifil II) against adjacent enamel erosion^31^. However, the results are not comparable, because the erosive cycling is totally different. In the mentioned study specimens were immersed in 0.3% citric acid, 4 times per day with an interval of 1 h in artificial saliva, for 5 days. On the other hand, in the present study specimens were immersed in 0.5% citric acid, 6 times per day with an interval of 2 h in artificial saliva, for 5 days. In addition, they measured only one area of enamel and the present study measured 5 different distances from the restoration margin. This was the novelty and the strength of our study since it was possible to clarify that the protective effect is restricted to enamel very close to the material. However, this effect should not be ignored, since the frequent acid exposure could affect the margins of adhesive restorations, what could maybe allow the flow of fluids through the adhesive interface^41^.

A few previous in vitro and in situ studies found no difference on the enamel loss prevention around different types of materials (amalgam, composite resin, and GIC) against erosion using profilometry and hardness measures^19,28^. The contradictory results, compared to the present study, could be due to the differences in the profile measurement method, materials composition, and the erosive protocol. On those studies, the profile was assessed at around 1.5 mm from the material margin^19^, probably missing the potential protective effect of bioactive materials, which was shown in our study. Our findings are in agreement with other studies that found less enamel loss adjacent to GICs^12,13^. The present study found the best protective effect for GHRSe, but the effect of the modified GIC (RMGIe) was similar to that of the composite resin group (CRe-Z350), which was not expected^19,41^. In contrast, another study found that the resin-modified GIC (Fuji II LC) was the only material able to protect the surrounding enamel against the erosive and erosive-abrasive challenges^39^, confirming that significant variation exists among materials within the same category, depending on factors such as the nature and size of the filler particles^11^ and the presence of resin. The resin-modified GIC exhibits in general a short-term weaker fluoride release as compared with the conventional GIC^31^, which might explain the decreased ability to protect the adjacent enamel against erosive challenge.

The specimens subjected to erosion and abrasion had no difference in enamel loss among the studied materials. Probably the ions released by the S-PRG-based composite groups and the fluoride released by the GIC groups did not sufficiently increase the enamel mechanical resistance to abrasion. It is known that even highly concentrated polyvalent metal fluorides have limited protective effect, and depending on the abrasion assay, the mechanical impact overcomes the chemical protection^42^.

The results of the present study should be considered with caution since the study was conducted in vitro with the limitation of using bovine enamel and artificial saliva. In addition further studies are needed evaluating the resistance of the S-PRG-based composite resins against occlusal loading. On the other hand, it was the first study to analyze wear at different distances from the restoration margin. The S-PRG-based composite resins presented higher resistance to erosive and/or abrasive wear than GICs and promoted similar protection against erosion alone of enamel very near the restoration. Clinically, this SPRG’s technology protective effect could be potentially beneficial for restorative treatment on patients with erosive tooth wear where mechanical forces, like abrasion can be controlled or diminished, but the erosive factor from chemical sources are not avoidable.

## Conclusion

S-PRG-based composites are resistant to erosive and abrasive challenges and are able to prevent wear of the enamel that is very near to the restoration margins when subjected to erosion, but not to erosion and abrasion.

## Data Availability

The datasets generated and/or analyzed during the current study are available from the corresponding author on reasonable request.
